# 5-Nitro-2-trifluoro­methyl-1*H*-benzimidazole monohydrate

**DOI:** 10.1107/S1600536812017060

**Published:** 2012-04-21

**Authors:** Ming-Liang Liu

**Affiliations:** a College of Chemistry and Chemical Engineering, Southeast University, Nanjing 210096, People’s Republic of China

## Abstract

In the crystal structure of the title compound, C_8_H_4_F_3_N_3_O_2_·H_2_O, the main mol­ecule and the water mol­ecule are linked by an N—H⋯O hydrogen bond. O—H⋯N, O—H⋯O and C—H⋯O hydrogen bonds further link the mol­ecules into sheets.

## Related literature

The title compound was studied as part of a search for ferroelectric complexes. For background to ferroelectric complexes, see: Zhang *et al.* (2009 ▶, 2010 ▶); Ye *et al.* (2009 ▶). For related structures, see: Liu (2011*a* ▶,*b* ▶).
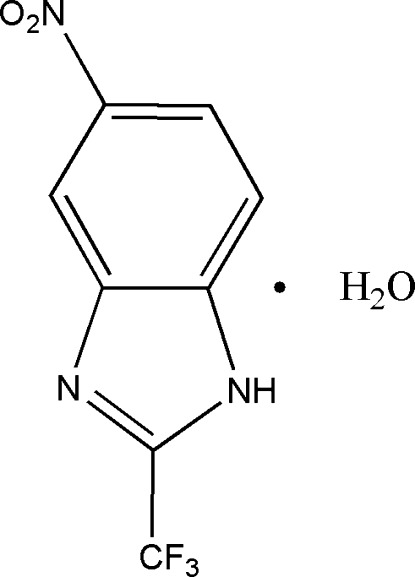


## Experimental

### 

#### Crystal data

C_8_H_4_F_3_N_3_O_2_·H_2_O
*M* *_r_* = 249.16
Monoclinic, 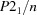

*a* = 7.6209 (15) Å
*b* = 10.393 (2) Å
*c* = 13.093 (3) Å
β = 97.63 (3)°
*V* = 1027.9 (4) Å^3^
*Z* = 4
Mo *K*α radiation
μ = 0.16 mm^−1^
*T* = 293 K
0.36 × 0.32 × 0.28 mm

#### Data collection

Rigaku Mercury2 diffractometer
Absorption correction: multi-scan (*CrystalClear*; Rigaku, 2005 ▶) *T*_min_ = 0.903, *T*_max_ = 0.921
10402 measured reflections
2344 independent reflections
1451 reflections with *I* > 2σ(*I*)
*R*_int_ = 0.051

#### Refinement

*R*[*F* ^2^ > 2σ(*F* ^2^)] = 0.071
*wR*(*F* ^2^) = 0.221
*S* = 1.05
2344 reflections
154 parameters
H-atom parameters constrained
Δρ_max_ = 0.59 e Å^−3^
Δρ_min_ = −0.32 e Å^−3^

### 

Data collection: *CrystalClear* (Rigaku, 2005 ▶); cell refinement: *CrystalClear*; data reduction: *CrystalClear*; program(s) used to solve structure: *SHELXS97* (Sheldrick, 2008 ▶); program(s) used to refine structure: *SHELXL97* (Sheldrick, 2008 ▶); molecular graphics: *PLATON* (Spek, 2009) ▶; software used to prepare material for publication: *SHELXTL* (Sheldrick, 2008 ▶).

## Supplementary Material

Crystal structure: contains datablock(s) I, global. DOI: 10.1107/S1600536812017060/go2048sup1.cif

Structure factors: contains datablock(s) I. DOI: 10.1107/S1600536812017060/go2048Isup2.hkl

Supplementary material file. DOI: 10.1107/S1600536812017060/go2048Isup3.cml

Additional supplementary materials:  crystallographic information; 3D view; checkCIF report

## Figures and Tables

**Table 1:** Hydrogen-bond geometry (Å, °)

*D*—H⋯*A*	*D*—H	H⋯*A*	*D*⋯*A*	*D*—H⋯*A*
N1—H1*A*⋯O3	0.86	1.90	2.740 (3)	166
O3—H3*A*⋯N2^i^	0.92	1.96	2.872 (3)	169
O3—H3*B*⋯O2^ii^	0.76	2.30	3.050 (4)	170
C6—H6⋯O1^ii^	0.93	2.55	3.380 (4)	148

Symmetry codes: (i) 
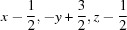
; (ii) 
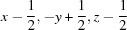
.

**Fig. 1.**
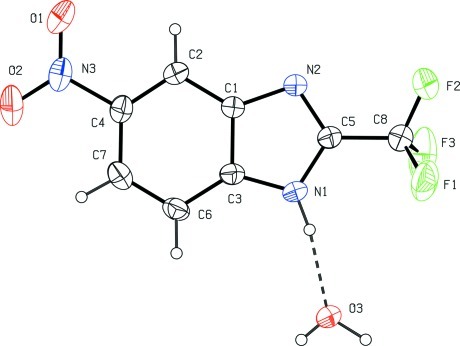
The molecular structure of the title compound, showing the atomic numbering scheme with 30% probability displacement ellipsoids.

**Fig. 2.**
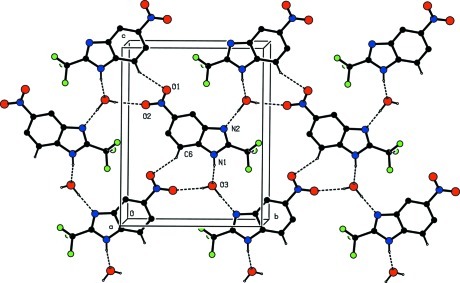
View of the sheet formed by the hydrogen bonding. Hydrogen atoms not involved in the hydrogen bonding are omitted for clarity. The labelled C, N and O atoms lie in the asymmetric unit.

## supplementary crystallographic information

### Comment

Recently much attention has been devoted to finding ferroelectric complexes. Ferroelectric materials that exhibit reversible electric polarization in response to an external electric field have found many applications such as nonvolatile memory storage, electronics and optics. The freezing of a certain functional group at low temperature forces significant orientational motions of the guest molecules and thus induces the formation of the ferroelectric phase. (Zhang *et al.* 2009; Ye *et al.* 2009; Zhang *et al.* 2010.). The title compound has been synthesized to investigate these properties.

The asymmetric unit of the title compound consists of one 5-nitro-2-trifluoromethylbenzimidazole molecule and one water molecule, (Figure 1), linked by the N1···H1A···O3 hydrogen bond, Table 1. The O3—H3A···.N2(x-1/2, -y+3/2, z-1/2), O3—H3B···O2(x-1/2, -y+1/2, z-1/2) and C6—H6···O1(x-1/2, -y+1/2, z-1/2), Table 1, intermolecular hydrogen bonds link the molecules to form sheets.

### Experimental

5-nitro-2-trifluoromethylbenzimidazole was dissolved in ethanol to give a solution without any precipitate while stirring at the ambient temperature. Single crystals suitable for X-ray structure analysis were obtained by the slow evaporation of the above solution after 5 days in air.

### Refinement

H atoms were placed in calculated positions (N—H = 0.86 Å; C—H = 0.93 Å and were assigned fixed [*U*_iso_ = 1.2*U*eq and allowed to ride. The H atoms bonding to the water O atom were found in difference Fourier map and fixed in the positions and allowed to ride with a fixed [*U*_iso_ = 1.5*U*eq. The final positions of the hydrogen atoms were checked on a difference Fourier map.

### Crystal data

**Table:**

C_8_H_4_F_3_N_3_O_2_·H_2_O	*Z* = 4
*M**_r_* = 249.16	*F*(000) = 504
Monoclinic, *P*2_1_/*n*	*D*_x_ = 1.610 Mg m^−^^3^
Hall symbol: -P 2yn	Mo *K*α radiation, λ = 0.71073 Å
*a* = 7.6209 (15) Å	θ = 3.4–27.5°
*b* = 10.393 (2) Å	µ = 0.16 mm^−^^1^
*c* = 13.093 (3) Å	*T* = 293 K
β = 97.63 (3)°	Block, colourless
*V* = 1027.9 (4) Å^3^	0.36 × 0.32 × 0.28 mm

### Data collection

**Table:**

Rigaku Mercury2 diffractometer	2344 independent reflections
Radiation source: fine-focus sealed tube	1451 reflections with *I* > 2σ(*I*)
Graphite monochromator	*R*_int_ = 0.051
CCD_Profile_fitting scans	θ_max_ = 27.5°, θ_min_ = 3.1°
Absorption correction: multi-scan (*CrystalClear*; Rigaku, 2005)	*h* = −9→9
*T*_min_ = 0.903, *T*_max_ = 0.921	*k* = −13→13
10402 measured reflections	*l* = −16→16

### Refinement

**Table:**

Refinement on *F*^2^	Primary atom site location: structure-invariant direct methods
Least-squares matrix: full	Secondary atom site location: difference Fourier map
*R*[*F*^2^ > 2σ(*F*^2^)] = 0.071	Hydrogen site location: inferred from neighbouring sites
*wR*(*F*^2^) = 0.221	H-atom parameters constrained
*S* = 1.05	*w* = 1/[σ^2^(*F*_o_^2^) + (0.1019*P*)^2^ + 0.6421*P*] where *P* = (*F*_o_^2^ + 2*F*_c_^2^)/3
2344 reflections	(Δ/σ)_max_ < 0.001
154 parameters	Δρ_max_ = 0.59 e Å^−^^3^
0 restraints	Δρ_min_ = −0.32 e Å^−^^3^

### Special details

**Table:**

Geometry. All esds (except the esd in the dihedral angle between two l.s. planes) are estimated using the full covariance matrix. The cell esds are taken into account individually in the estimation of esds in distances, angles and torsion angles; correlations between esds in cell parameters are only used when they are defined by crystal symmetry. An approximate (isotropic) treatment of cell esds is used for estimating esds involving l.s. planes.
Refinement. Refinement of *F*^2^ against ALL reflections. The weighted *R*-factor *wR* and goodness of fit *S* are based on *F*^2^, conventional *R*-factors *R* are based on *F*, with *F* set to zero for negative *F*^2^. The threshold expression of *F*^2^ > σ(*F*^2^) is used only for calculating *R*-factors(gt) *etc*. and is not relevant to the choice of reflections for refinement. *R*-factors based on *F*^2^ are statistically about twice as large as those based on *F*, and *R*- factors based on ALL data will be even larger.

### Fractional atomic coordinates and isotropic or equivalent isotropic displacement parameters (Å^2^)

**Table:**

	*x*	*y*	*z*	*U*_iso_*/*U*_eq_	
F1	0.7199 (4)	0.8890 (3)	0.31003 (18)	0.1071 (10)	
F2	0.7951 (5)	0.9620 (2)	0.4602 (3)	0.1403 (15)	
F3	0.5316 (4)	0.9177 (3)	0.4078 (3)	0.1320 (13)	
O1	0.9829 (5)	0.3172 (3)	0.7630 (3)	0.0971 (11)	
O2	0.8506 (5)	0.1639 (3)	0.6747 (3)	0.1086 (12)	
N1	0.6638 (3)	0.6404 (2)	0.38970 (18)	0.0471 (6)	
H1A	0.6092	0.6413	0.3279	0.057*	
N2	0.8035 (3)	0.7156 (2)	0.53963 (18)	0.0469 (6)	
N3	0.8922 (4)	0.2768 (3)	0.6865 (3)	0.0687 (9)	
C1	0.7950 (4)	0.5815 (3)	0.5434 (2)	0.0414 (7)	
C2	0.8576 (4)	0.4973 (3)	0.6230 (2)	0.0493 (7)	
H2	0.9157	0.5264	0.6857	0.059*	
C3	0.7071 (4)	0.5333 (3)	0.4496 (2)	0.0426 (7)	
C4	0.8283 (4)	0.3683 (3)	0.6032 (2)	0.0507 (8)	
C5	0.7237 (4)	0.7440 (3)	0.4469 (2)	0.0449 (7)	
C6	0.6781 (4)	0.4023 (3)	0.4317 (2)	0.0517 (8)	
H6	0.6194	0.3722	0.3695	0.062*	
C7	0.7404 (4)	0.3194 (3)	0.5103 (3)	0.0566 (8)	
H7	0.7242	0.2311	0.5018	0.068*	
C8	0.6956 (5)	0.8789 (3)	0.4077 (3)	0.0575 (8)	
O3	0.4481 (4)	0.6193 (2)	0.20564 (17)	0.0755 (9)	
H3A	0.4159	0.6728	0.1499	0.113*	
H3B	0.4169	0.5518	0.1909	0.113*	

### Atomic displacement parameters (Å^2^)

**Table:**

	*U*^11^	*U*^22^	*U*^33^	*U*^12^	*U*^13^	*U*^23^
F1	0.182 (3)	0.0760 (16)	0.0689 (15)	0.0252 (17)	0.0363 (17)	0.0271 (13)
F2	0.216 (4)	0.0534 (15)	0.126 (2)	−0.0328 (19)	−0.072 (2)	0.0178 (15)
F3	0.113 (2)	0.090 (2)	0.203 (3)	0.0460 (17)	0.059 (2)	0.063 (2)
O1	0.115 (3)	0.081 (2)	0.084 (2)	0.0147 (18)	−0.0250 (19)	0.0302 (17)
O2	0.141 (3)	0.0490 (17)	0.131 (3)	0.0068 (18)	−0.002 (2)	0.0273 (17)
N1	0.0566 (15)	0.0497 (15)	0.0322 (12)	−0.0006 (12)	−0.0049 (10)	−0.0002 (10)
N2	0.0561 (15)	0.0411 (13)	0.0406 (13)	−0.0014 (11)	−0.0048 (11)	−0.0018 (10)
N3	0.075 (2)	0.0540 (19)	0.077 (2)	0.0149 (16)	0.0096 (17)	0.0202 (16)
C1	0.0439 (15)	0.0405 (15)	0.0387 (14)	−0.0014 (12)	0.0009 (12)	−0.0026 (12)
C2	0.0520 (17)	0.0517 (18)	0.0412 (15)	−0.0007 (14)	−0.0047 (13)	0.0020 (13)
C3	0.0439 (15)	0.0479 (17)	0.0355 (14)	−0.0031 (12)	0.0030 (11)	−0.0035 (12)
C4	0.0540 (17)	0.0450 (17)	0.0532 (18)	0.0084 (14)	0.0077 (14)	0.0076 (14)
C5	0.0513 (16)	0.0460 (16)	0.0363 (14)	0.0009 (13)	0.0015 (12)	−0.0007 (12)
C6	0.0626 (19)	0.0472 (18)	0.0448 (16)	−0.0064 (14)	0.0050 (14)	−0.0128 (13)
C7	0.066 (2)	0.0400 (17)	0.065 (2)	−0.0043 (15)	0.0136 (16)	−0.0068 (15)
C8	0.067 (2)	0.0504 (19)	0.0530 (19)	0.0026 (16)	0.0013 (16)	0.0036 (16)
O3	0.115 (2)	0.0493 (13)	0.0507 (14)	0.0082 (13)	−0.0307 (14)	−0.0022 (11)

### Geometric parameters (Å, º)

**Table:**

F1—C8	1.320 (4)	C1—C3	1.410 (4)
F2—C8	1.287 (4)	C2—C4	1.378 (5)
F3—C8	1.313 (4)	C2—H2	0.9300
O1—N3	1.214 (4)	C3—C6	1.394 (4)
O2—N3	1.219 (4)	C4—C7	1.403 (5)
N1—C5	1.355 (4)	C5—C8	1.499 (4)
N1—C3	1.377 (4)	C6—C7	1.377 (5)
N1—H1A	0.8596	C6—H6	0.9300
N2—C5	1.317 (4)	C7—H7	0.9300
N2—C1	1.397 (4)	O3—H3A	0.9240
N3—C4	1.481 (4)	O3—H3B	0.7573
C1—C2	1.396 (4)		
			
C5—N1—C3	106.8 (2)	C7—C4—N3	118.6 (3)
C5—N1—H1A	126.6	N2—C5—N1	114.3 (3)
C3—N1—H1A	126.5	N2—C5—C8	123.5 (3)
C5—N2—C1	103.8 (2)	N1—C5—C8	122.1 (3)
O1—N3—O2	123.2 (3)	C7—C6—C3	117.0 (3)
O1—N3—C4	118.7 (3)	C7—C6—H6	121.5
O2—N3—C4	118.0 (4)	C3—C6—H6	121.5
C2—C1—N2	129.8 (3)	C6—C7—C4	119.9 (3)
C2—C1—C3	120.2 (3)	C6—C7—H7	120.1
N2—C1—C3	110.0 (2)	C4—C7—H7	120.1
C4—C2—C1	116.0 (3)	F2—C8—F3	106.7 (4)
C4—C2—H2	122.0	F2—C8—F1	108.4 (3)
C1—C2—H2	122.0	F3—C8—F1	103.5 (3)
N1—C3—C6	132.3 (3)	F2—C8—C5	113.4 (3)
N1—C3—C1	105.0 (2)	F3—C8—C5	112.3 (3)
C6—C3—C1	122.7 (3)	F1—C8—C5	111.9 (3)
C2—C4—C7	124.3 (3)	H3A—O3—H3B	108.4
C2—C4—N3	117.2 (3)		
			
C5—N2—C1—C2	−179.8 (3)	C1—N2—C5—N1	0.0 (3)
C5—N2—C1—C3	−0.1 (3)	C1—N2—C5—C8	178.3 (3)
N2—C1—C2—C4	179.8 (3)	C3—N1—C5—N2	0.1 (3)
C3—C1—C2—C4	0.2 (4)	C3—N1—C5—C8	−178.2 (3)
C5—N1—C3—C6	179.5 (3)	N1—C3—C6—C7	−180.0 (3)
C5—N1—C3—C1	−0.2 (3)	C1—C3—C6—C7	−0.3 (5)
C2—C1—C3—N1	179.9 (3)	C3—C6—C7—C4	0.1 (5)
N2—C1—C3—N1	0.2 (3)	C2—C4—C7—C6	0.3 (5)
C2—C1—C3—C6	0.1 (4)	N3—C4—C7—C6	179.4 (3)
N2—C1—C3—C6	−179.5 (3)	N2—C5—C8—F2	17.5 (5)
C1—C2—C4—C7	−0.5 (5)	N1—C5—C8—F2	−164.4 (3)
C1—C2—C4—N3	−179.6 (3)	N2—C5—C8—F3	−103.5 (4)
O1—N3—C4—C2	−7.8 (5)	N1—C5—C8—F3	74.6 (4)
O2—N3—C4—C2	172.1 (3)	N2—C5—C8—F1	140.6 (3)
O1—N3—C4—C7	173.0 (3)	N1—C5—C8—F1	−41.3 (4)
O2—N3—C4—C7	−7.0 (5)		

### Hydrogen-bond geometry (Å, º)

**Table:**

*D*—H···*A*	*D*—H	H···*A*	*D*···*A*	*D*—H···*A*
N1—H1*A*···O3	0.86	1.90	2.740 (3)	166
O3—H3*A*···N2^i^	0.92	1.96	2.872 (3)	169
O3—H3*B*···O2^ii^	0.76	2.30	3.050 (4)	170
C6—H6···O1^ii^	0.93	2.55	3.380 (4)	148

Symmetry codes: (i) *x*−1/2, −*y*+3/2, *z*−1/2; (ii) *x*−1/2, −*y*+1/2, *z*−1/2.

## References

[bb1] Liu, M.-L. (2011*a*). *Acta Cryst.* E**67**, o2821. 10.1107/S1600536811039298 PMC3247562 22219867

[bb2] Liu, M.-L. (2011*b*). *Acta Cryst.* E**67**, o3473. 10.1107/S1600536811048811 PMC3239099 22199947

[bb3] Rigaku (2005). *CrystalClear* Rigaku Corporation, Tokyo, Japan.

[bb4] Sheldrick, G. M. (2008). *Acta Cryst.* A**64**, 112–122. 10.1107/S0108767307043930 18156677

[bb5] Spek, A. L. (2009). *Acta Cryst.* D**65**, 148–155. 10.1107/S090744490804362X PMC2631630 19171970

[bb6] Ye, H. Y., Fu, D. W., Zhang, Y., Zhang, W., Xiong, R. G. & Huang, S. P. (2009). *J. Am. Chem. Soc.* **131**, 42–43. 10.1021/ja808331g 19128170

[bb7] Zhang, W., Chen, L. Z., Xiong, R. G., Nakamura, T. & Huang, S. P. (2009). *J. Am. Chem. Soc.* **131**, 12544–12545. 10.1021/ja905399x 19685869

[bb8] Zhang, W., Ye, H. Y., Cai, H. L., Ge, J. Z., Xiong, R. G. & Huang, S. P. (2010). *J. Am. Chem. Soc.* **132**, 7300–7302. 10.1021/ja102573h 20459097

